# Transcatheter Mitral Valve Replacement and Thrombosis: A Review

**DOI:** 10.3389/fcvm.2021.621258

**Published:** 2021-06-04

**Authors:** Guido Ascione, Paolo Denti

**Affiliations:** Department of Cardiac Surgery, IRCCS San Raffaele Hospital, Vita-Salute San Raffaele University, Milan, Italy

**Keywords:** transcatheter mitral valve replacement, thrombosis, vitamin K antagonists, non-vitamin K antagonist oral anticoagulants, mitral valve regurgitation

## Abstract

Mitral regurgitation is the most prevalent form of moderate or severe valve disease in developed countries. Surgery represents the standard of care for symptomatic patients with severe mitral regurgitation, but up to 50% of patients are denied surgery because of high surgical risk. In this context, different transcatheter options have been developed to address this unmet need. Transcatheter mitral valve replacement (TMVR) is an emergent field representing an alternative option in high complex contexts when transcatheter mitral valve repair is not feasible or suboptimal due to anatomical issues. However, TMVR is burdened by some device-specific issues (device malposition, migration or embolization, left ventricular outflow tract obstruction, hemolysis, thrombosis, stroke). Here we discuss the thrombotic risk of TMVR and current evidence about anticoagulation therapy after TMVR.

## Introduction

Mitral regurgitation (MR) is the most prevalent form of moderate or severe valve disease in developed countries. MR may be either primary (due to valve leaflets or subvalvular apparatus dysfunction) or secondary to left ventricular remodeling in the context of chronic ischemic heart disease or primary myocardial disease. The most common causes of primary MR in industrialized countries are mitral valve prolapse, rheumatic heart disease, and endocarditis (1).

Surgery (either mitral valve repair or mitral valve replacement) represents the standard of care to treat severe symptomatic MR. However, almost 50% of patients are denied surgery because of high surgical risk [impaired left ventricular ejection fraction (LVEF), older age, comorbidities] (2). As a consequence, different transcatheter therapeutic treatments have been developed in the last years to address this unmet need.

Transcatheter mitral valve replacement (TMVR) is an emerging field representing an alternative option to treat severe symptomatic MR in high complex contexts when transcatheter mitral valve repair is not feasible or suboptimal due to anatomical issues.

**Figure 1:**
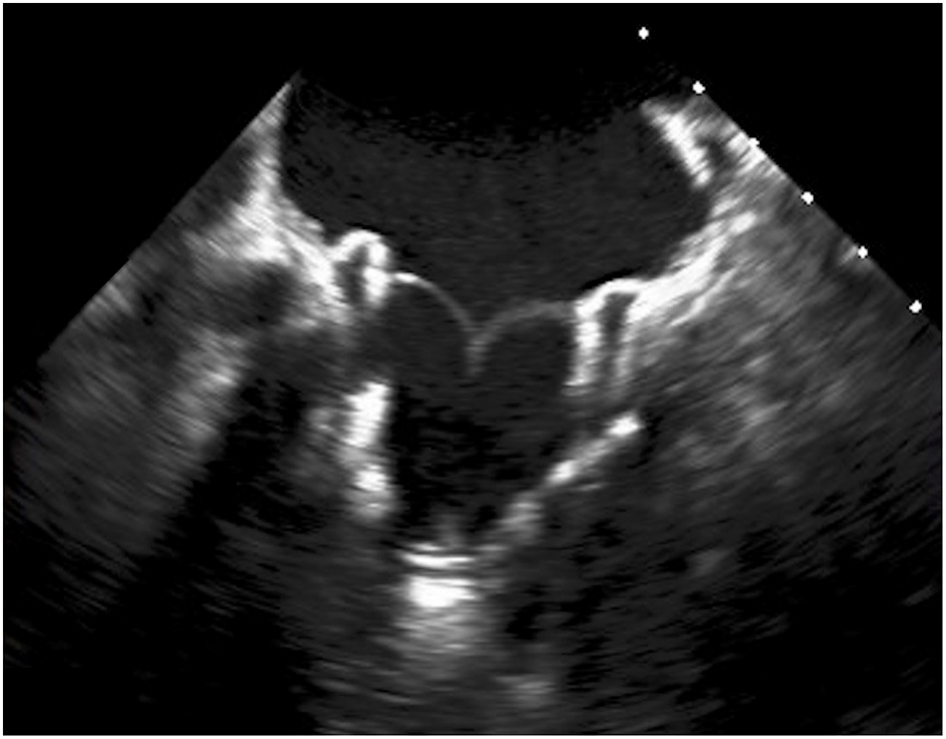
Transesophageal echocardiography showing Tendyne^TM^ valve (Abbott) after deployment.

**Figure 2:**
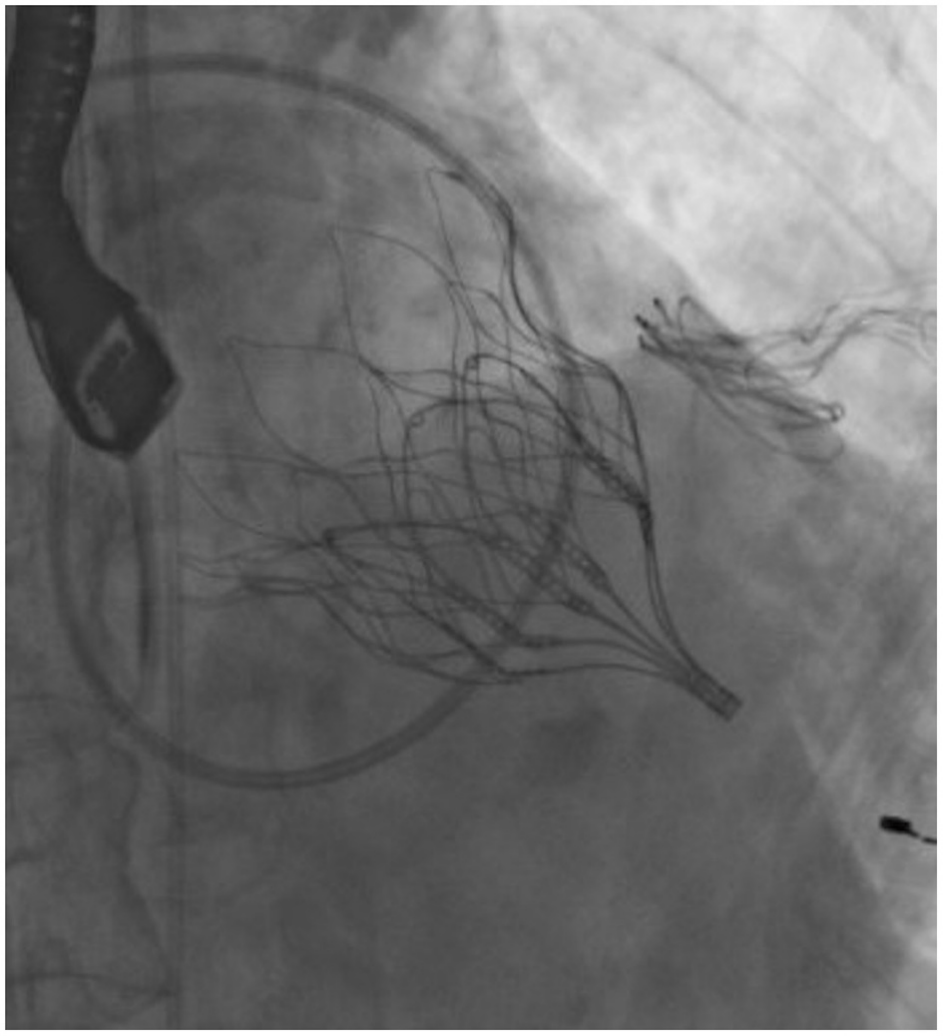
Fluoroscopy showing Tendyne^TM^ valve (Abbott) after deployment.

Few devices are under clinical evaluation (an example in Figures 1, 2), while the majority of them are still undergoing pre-clinical trials, and few data are available on mid- and long-term results (3).

However, TMVR is burdened by some device-specific risks, linked with both the complexity of mitral valve anatomy [device malposition, migration or embolization, left ventricular outflow tract (LVOT) obstruction] and the design of the devices themselves (hemolysis, thrombosis, stroke) (3).

Here we discuss the thrombotic risk of TMVR, as compared to transcatheter aortic valve replacement (TAVR) and surgical bioprosthesis implantation, and the antithrombotic strategies after TMVR.

## Prosthetic Valve Thrombosis

### Pathophysiology of Prosthetic Valve Thrombosis

Prosthetic valve thrombosis (PVT) is defined as thrombus formation on the exogenous valve structure, associated with valve dysfunction and, potentially, thromboembolic phenomena (4).

PVT is a multifactorial process involving surface-, hemodynamic-, and hemostasis-related factors (4). The so-called Virchow's triad accurately describes the three main elements that may lead to endovascular/intracardiac thrombosis (5, 6): blood flow alterations (stasis, turbulence), hypercoagulability (both congenital or acquired), and endothelial injury/dysfunction (7).

Following any cardiac device implantation, thrombosis may occur in two different ways: either by direct activation of coagulation cascade on the exogenous device surface or, indirectly, as a result of hemodynamic changes induced by the device itself (device-related thrombosis) (7).

For example, cases of early left atrial appendage thrombosis (8) or left ventricular thrombosis (9) are reported after successful Mitraclip implantation, and these data have been related to the role Mitraclip-induced altered hemodynamics may have in increasing thrombogenicity. Obviously, also TMVR devices carry a similar risk, considering the structural complexity they need to safely anchor on the saddle-shaped mitral valve annulus.

Thromboembolic risk is further increased by underlying cardiac pathologies (i.e., heart failure or atrial fibrillation), leading to disturbances in endothelial function, blood flow, and blood composition (7).

Furthermore, any exogenous device implanted in the mitral position is affected by higher thrombotic risk if compared with the aortic position (4), as shown by TAVR devices implanted in mitral position in the context of surgical mitral valve repair failure [Valve-in-Ring (ViR)] or surgical bioprosthesis dysfunction [Valve-in-Valve, (ViV)]. Higher rates of valve thrombosis have been observed in these situations (up to 15%) (10, 11), and this phenomenon is probably related to low-flow conditions existing in the left atrium and the left ventricular inflow tract if compared to the LVOT and the aorta.

### Clinical Presentation of Valve Thrombosis

Patients with valve thrombosis may present with signs or symptoms of heart failure (progressive dyspnea, ankle swelling) or peripheral embolization (i.e., stroke, acute limb ischemia, acute mesenteric ischemia, acute ischemic kidney injury). Alternatively, thrombosis may be an incidental finding during follow-up echocardiography in asymptomatic patients (12). In case of delayed diagnosis, fulminant cardiogenic shock may occur (13).

Obviously, other causes of valve dysfunction (such as valve deterioration or endocarditis) and other causes of embolization (i.e., left atrial thrombus in the context of atrial fibrillation, endocarditis) should be ruled out when dealing with patients with new onset heart failure (HF) or peripheral embolization stigmata (4).

### Valve Thrombosis Diagnostic Tools

In a patient with a prosthetic valve presenting with new onset HF symptoms or thromboembolism of unknown origin, the purpose of cardiovascular imaging is to rule out valve thrombosis and eventually assess its severity, etiology, and hemodynamic consequences.

**Figure 3:**
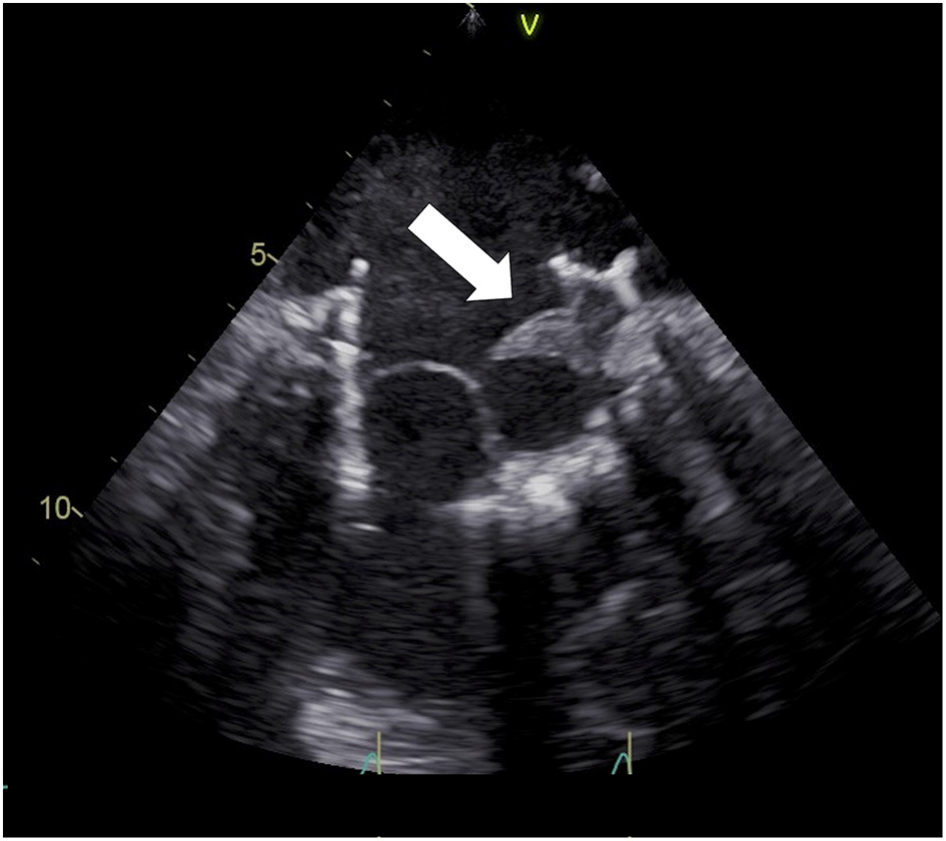
Transesophageal echocardiography showing Tiara (Neovasc) anterior leaflet thrombosis.

First-line diagnostic test is transthoracic echocardiography, usually showing increased transvalvular gradients, reduced leaflet mobility, abnormal intravalvular regurgitation, or images suggestive for thrombi (high-density lesions, usually located on the atrial side of the valve, originally appearing on the valve ring and then moving toward leaflets) (14). Transesophageal echocardiography should be considered as an adjunctive tool if transthoracic echocardiography is suboptimal or in any case of etiological doubt (13) (Figure 3). When echocardiography is inconclusive, cardiac CT scan may help to better assess prosthetic valve functioning. It is a useful tool, for example, to analyze leaflet motion and distinguish valve thrombosis from valve deterioration (3).

## Bioprosthesis and Thromboembolic Risk

### Thrombotic Risk After Surgical Mitral Valve Replacement

The long-term experience with surgical bioprosthesis has led to robust data about thromboembolic susceptibility of these devices. However, the reported rates of thrombosis are highly variable and surely underestimate the burden of the phenomenon because valve imaging is not routinely performed during follow-up and valve thrombosis is sometimes subclinical, with spontaneous resolution before symptoms appear (4). The annual rate of surgical bioprosthesis thrombosis, considering devices implanted both in the aortic and mitral position, ranges between 0.03 and 0.74% (11). The risk of thrombosis is higher in the first 3 months after surgery (when the neointimal layer is not yet formed on valve leaflets), and it appears to be influenced also by the type of the prosthetic valve, being stentless and pericardial valves less thrombogenic than porcine stented ones (4).

### Thrombotic Risk After TAVR

Analysis based on retrospective data estimated an incidence of valve thrombosis after TAVR of 0.6–2.8% (15, 16). Furthermore, cardiac CT studies conducted on TAVR patients led to the identification of the so-called “subclinical thrombosis,” namely, an incidental radiographic finding of thrombus stratification on valve leaflets, in the absence of any symptom or echocardiographic sign of valve dysfunction. It is still an object of debate if subclinical thrombosis is a precursor of a proper one, and further studies are necessary to fully understand its implications on valve durability and risk of thromboembolic events (17, 18).

The higher incidence of valve thrombosis in TAVR if compared with surgical aortic valve replacement can be partially explained considering that, during transcatheter aortic valve replacement, native aortic valve leaflets remain in place and are pushed against Valsalva sinuses. Both the endothelial damage to native leaflets and the turbulent flow in the neo-sinuses have been proposed as a thrombotic trigger, especially in the first 3 months after implantation, when endothelialization of the valve is not yet complete (19).

### Thrombotic Risk After TMVR

TMVR is an emerging field, and so few data are available on the thrombogenicity of transcatheter mitral prosthesis.

New transcatheter mitral devices, specifically designed to be allocated in the saddle-shaped, dynamic mitral annulus, are under investigation as an option to treat severe mitral regurgitation in patients with a native mitral valve but high surgical risk (3).

Table 1 describes available data on thrombotic risk of these devices.

**Table 1:** Available data on thrombotic risk of currently developed devices for TMVR in native mitral valve disease (3).

	**Reported device thrombosis (*****n*****, %)**	
	**Procedural and 30 days follow-up**	**Midterm follow-up^**a**^**
AltaValve (4C medical technologies)	0/1 (0)	NA
Caisson (LivaNova)	NA	NA
CardiAQ (Edwards lifesciences)	NA	NA
CardioValve (Cardiovalve)	NA	NA
Fortis (Edwards lifesciences)	1/13 (7.7)	NA
HighLife (HighLife SAS)	1/15 (6.6)	NA
Intrepid (Medtronic)	0/50 (0)	0/50 (0)
MValve System (MValve technologies)	NA	NA
Tiara (Neovasc)	NA	NA
Sapien M3 (Edwards lifesciences)	NA	NA
Tendyne (Abbott)	1/100 (1)	6/100 (6)
Global Cohort	3/179 (1.7)	6/150 (4)

a *Mean follow-up was 10.1 ± 0.3 months, ranging from 3 to 24 months (3)*.

Interestingly, a higher rate of thrombosis is reported (from 6 to 8%) if compared with surgical mitral bioprosthesis and TAVR (3, 20).

Some reasons may explain such a finding:

- During TMVR, like during TAVR, mitral valve native tissue is not removed, differently from mitral valve surgical replacement. The endocardial damage induced by valve positioning and the degeneration of native valve leaflets may trigger thrombus formation.
- Transcatheter mitral devices are equipped with bulky anchoring systems, which may trigger thrombosis, and this is particularly true in low-flow left ventricular conditions (as in patients with low LVEF).
- The 3D relationship between the transcatheter valve and the mitral valve annulus, with some gaps due to the imperfect sealing, may create prothrombotic flow turbulence.
- The transapical approach, often used to deploy the valve, modifies ventricular geometry and is thus associated with a higher risk of thrombosis.
- A significant proportion of patients enrolled in the feasibility studies of these devices (from 30 to 60%) (21–23) were affected by atrial fibrillation, which enhances itself the risk of thrombi formation, considering the associated left atrial low-flow status.

Similar considerations are applicable also to TMVR in patients with failure of surgical annuloplasty (ViR) or degeneration of surgical bioprosthesis (ViV) who are deemed too high risk to undergo REDO surgery. In this context, transcatheter devices originally designed for TAVR have been mainly used.

Different rates of thrombosis are reported, ranging from 1.6 to 7% (24, 25). ViV procedures appeared to be more thrombogenic than ViR, especially if performed in previously surgically implanted stented porcine valves. Lower rates of thrombosis were reported in patients with previously implanted bovine pericardial valves, in accordance with available data on surgically implanted bioprosthesis and TAVR ViV (4, 16).

ViR thrombosis may be explained by the low-flow status existing at the interface between the transcatheter valve, the surgical ring (in the presence of even trivial perivalvular leaks due to imperfect valve sealing), and the native mitral valve apparatus. In ViV thrombosis, on the other hand, degenerated bioprosthesis calcifications, fibrosis, and tear may play a role in triggering thrombus formation (26).

TMVR with valves originally designed for TAVR has been also used to treat MR in patients with severe mitral annulus calcification (ViMAC). This anatomical finding increases surgical risk at the time of mitral valve replacement, being annular decalcification associated with both cardiac rupture at the atrioventricular junction and circumflex artery injury, while less aggressive debridement may cause severe periprosthetic mitral regurgitation (27).

No cases of thrombosis were reported in most of the studies about ViMAC (24, 26), while Guerrero et al. (28) reported a thrombosis rate of 1.8% at 1-year follow-up. Interestingly, a higher rate of thrombosis was found by Urena et al. (25) in a study conducted on 27 patients (11.1% at 30 days). All cases were treated by oral vitamin-K antagonist (VKA), with thrombosis resolution.

To date, no data are available about subclinical thrombosis in TMVR population.

## Antithrombotic Options After TMVR

The optimal antithrombotic treatment after TMVR remains controversial, as few data are available on long-term follow-up of these patients.

Current guidelines from the American Heart Association/American College of Cardiology suggest the use of anticoagulation with VKA to achieve a target INR of 2.5 for at least 3 months and for as long as 6 months after surgical bioprosthetic MVR in patients at low risk of bleeding and without other indications for anticoagulation. Anticoagulation is intended to reduce the risk of thromboembolism before the valve is fully endothelialized. Lifelong single antiplatelet agent (usually aspirin) is then suggested after discontinuation of anticoagulant therapy (29).

Similarly, the European Society of Cardiology/European Association of Cardiothoracic Surgery recommends oral anticoagulation with VKA for at least 3 months after surgical implantation of a mitral valve bioprosthesis (30).

Anticoagulation for a tissue prosthesis is supported by reports of valve thrombosis in patients undergoing bioprosthetic valve replacement (24).

Early experience with TMVR suggests a higher risk or thrombosis if compared with TAVR and surgical mitral valve replacement and a lower rate of thrombotic events in patients treated by VKA (20, 24, 31). It is thus reasonable to use some anticoagulant therapy in these patients.

**Figure 4:**
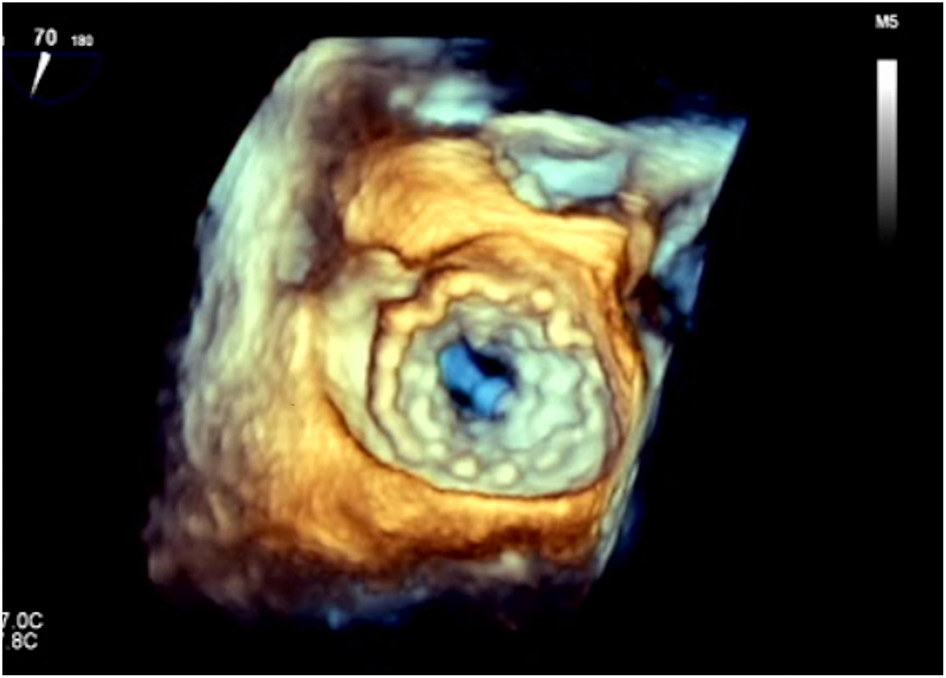
3D transesophageal echocardiography showing the atrial side of a Tendyne^TM^ valve (Abbott) after deployment. It exhibits the huge artificial anchoring surface of these devices.

However, no consensus is available on the duration of anticoagulation after TMVR. Thrombogenicity of the bulky transcatheter valves (Figure 4) may suggest prescribing lifelong anticoagulant therapy. On the other hand, TMVR population is mainly constituted by frail patients (with multiple comorbidities) at increased risk of bleeding during antithrombotic therapy. The optimal target INR and the possible association of an antiplatelet drug should be tailored on the structural characteristics of the implanted device and patient-specific risk factors [also considering the high incidence of atrial fibrillation in TMVR population both at baseline and after valve implantation (20, 21, 31)]. In patients with high bleeding risk (i.e., history of previous hemorrhage during anticoagulant therapy), the adoption of only an antiplatelet therapy may be reasonable.

Recently, new data are emerging about the role non-vitamin K antagonist oral anticoagulants (NOACs) may have in preventing thrombosis in patients with valvular heart diseases (32). Few randomized trials (33, 34) and meta-analysis (35) are available about the use of NOAC in patients with atrial fibrillation and biological prosthesis, both in the aortic and mitral position. They show, even after TAVR (35), similar rates of stroke and major bleeding if compared with VKA. NOAC may thus represent a promising alternative in TMVR patients.

In any case, a strict echocardiographic follow-up is imperative to detect early sign of valve dysfunction or thrombosis and to modify the anticoagulant therapy accordingly.

Further studies are required toward a progressive standardization of antithrombotic protocols in TMVR patients.

## Current Limitations and Future Directions

To date, only few data are available on TMVR outcomes, and they mainly come from observational studies. Thus, further prospective analyses are needed to address some unanswered questions about the thrombotic risk of these devices, the ideal duration of anticoagulation therapy, and the usefulness of antiplatelet drug association. There is also an unmet need to identify predictors of valve thrombosis, eventually outlining the pathophysiological differences between the thrombotic pathways of different devices (TMVR in the native mitral valve, ViV, ViR, ViMAC).

Furthermore, the identification of new materials, with less significant thrombotic properties, may help to develop valves associated with a lower rate of thromboembolic events. Some polymers are under pre-clinical investigation and might be applicable in the future to both transcatheter and surgical MVR (36). Finally, the role NOAC may have in TMVR population has to be fully investigated.

## Conclusions

TMVR is an emerging and promising treatment option for patients with severe symptomatic MR deemed too high risk to undergo conventional surgery. The optimal antithrombotic strategy is still an object of debate, but available data suggest a relevant risk of early and late thrombosis. Thus, a patient-tailored anticoagulation treatment is necessary, weighting accurately thromboembolic and bleeding risk in such a frail population.

## Author Contributions

GA: conceptualization, resources, formal analysis, writing—original draft, and visualization. PD: writing—review and editing, validation, supervision, and project administration. Both authors contributed to the article and approved the submitted version.

## Conflict of Interest

The authors declare that the research was conducted in the absence of any commercial or financial relationships that could be construed as a potential conflict of interest.
